# Prevalence and associated factors of anemia among postpartum mothers in public health facilities in Ethiopia, 2024: a systematic review and meta-analysis

**DOI:** 10.1186/s12884-024-06525-9

**Published:** 2024-04-27

**Authors:** Gebeyehu Lakew, Amlaku Nigusie Yirsaw, Alemshet Yirga Berhie, Asnake Gashaw Belayneh, Solomon Ketema Bogale, Eyob Getachew, Getnet Alemu Andarge, Kedir Seid, Eyob Ketema Bogale

**Affiliations:** 1 Health Promotion and Communication Department, School of public health, College of medicine and health sciences, Gondar University, Gondar, Ethiopia; 2 Nursing department, college of medicine and health science, Bahir Dar University, Bahir Dar, Ethiopia; 3 Department of emergency and critical care nursing, College of Medicine and Health Science, Bahir Dar University, Bahir Dar, Ethiopia; 4 Department of Nutrition, Antsokiya Gemza wereda Health Office, North Shoa, North East, Ethiopia; 5 Bati Primary Hospital, Oromia Special Zone, North Shoa, North Central, Ethiopia; 6 Health Promotion and Behavioral science department, school of public health, College of medicine and health science, Bahir Dar University Bahir Dar, Ethiopia

**Keywords:** Anemia, Hemoglobin, Postpartum women, Ethiopia

## Abstract

**Background:**

Postpartum anemia, characterized by hematocrit or hemoglobin levels below the defined cutoff point (< 11gm/dl or hematocrit < 33%), is a prevalent global issue. It serves as an indirect contributor to maternal mortality and morbidity. Mothers in the postpartum period experience diminished quality of life, impaired cognitive function, emotional instability, and an increased risk of postpartum depression due to anemia. Additionally, infants of affected mothers may face challenges such as insufficient breast milk supply and a lack of proper care. Examining the combined prevalence and factors associated with postpartum anemia is crucial for addressing maternal health risks and complications during the postnatal phase attributed to anemia.

**Objective:**

The study aimed to synthesize the existing literature on the prevalence and associated factors of postpartum anemia in public health facilities of Ethiopia, in 2024.

**Methods:**

The study was conducted by searching through the Google Scholar, PubMed, and Cochrane Library search engines. The search utilized keywords and MeSH terms such as anemia, low hemoglobin, postpartum, postnatal women, and Ethiopia. The collected data underwent analysis and comparison with the WHO criteria to determine if it met the threshold for declaring a public health concern. Heterogeneity was evaluated through the Cochran Q test and I2 statistics. Prevalence and odds ratio estimations were performed using a random-effects model with a 95% confidence interval.

**Result:**

Four studies were included in this systematic review and meta-analysis. The overall pooled prevalence of anemia among postpartum women in Ethiopia was 69% (95% CI: 60- 77%).Lack of formal education(OR = 3.5;CI:2.639,4.408),Low Pre-delivery hemoglobin (OR = 4.2;CI: 1.768–6.668), Postpartum women < 4 ANC visit (OR = 2.72; 95% CI:2.14,3.3 ),history of post partum hemorrhage (OR = 2.49; CI: 1.075–3.978),history of Forceps/vacuum delivery(OR = 3.96; CI:2.986–4.947), Poor iron and folic acid adherence (OR = 2.8;95% CI:2.311,3.297), C/S (OR = 4.04; 95% CI: 3.426,4.671),lower dietary diversity (OR = 4.295% CI:1.768,6.668) were significantly associated postpartum anemia.

**Conclusion:**

Postpartum women in Ethiopia continue to face a considerable public health challenge in the form of anemia. Consequently, there is a pressing need for the government to formulate comprehensive, multi-sectorial policies and strategies. These initiatives should be designed to address the substantial regional disparities influenced by interconnected factors, with the aim of reducing the prevalence of anemia among postpartum women in Ethiopia.

## Background

Anemia is characterized by a drop in red blood cell mass (RBC mass) or a low hemoglobin (Hb) level in comparison to the normal reference range [1]. When hemoglobin levels are less than 11 gm/dl at one week postpartum and less than 12 gm/dl at eight weeks postpartum, postpartum anemia—a persistent iron deficiency occurs [2].

Women who were nursing and had hemoglobin levels ≥ 12 g/dl were regarded as having a normal value. Mild anemia is defined as hemoglobin levels 11–11.9 g/dl, whereas moderate and severe anemia is defined as hemoglobin values 8–10.9 g/dl and < 8 g/dl, respectively [3]. Within the first 24 h following birth, hemoglobin concentration is reduced due to hemodynamic changes, fluid loss, and blood loss. But increases 48 h later and takes 7 days to reach the non-pregnant level [4].

Anemia afflicted 613 million (33%) of all women of reproductive age worldwide in 2016, with Asia and Africa having higher rates than the other two continents combined [4]. Anemia prevalence among new mothers varies from 10 to 30% in wealthy nations and 50–80% in underdeveloped nations; it is lower in Kenya (16.4%) and higher in south Rajasthan (90.68%) [5, 6]. Ethiopia’s 2020 objective was reduce anemia in the reproductive age group from 19.3 to 12%, but it resulted in an increase in anemia burden to 24%. In particular, the percentage of lactating mothers increased from 18.6% in 2011 to 28.6% in 2016 [7, 8].

The majority of maternal deaths worldwide happen in the postpartum phase; yet, several Sub-Saharan African nations, including Ethiopia, have a disproportionately higher burden of these deaths. Postpartum anemia is linked to postpartum depression, exhaustion, poor cognitive function, and disrupted mother-infant attachment. It affects up to 80% of women in low-income and rural communities and up to 50% of women in Europe and the US [9]. The postnatal period is the most crucial yet most ignored time in mothers’ and babies’ lives, according to the World Health Organization (WHO) [10]. The annual report from the Ethiopian Ministry of Health states that over 70% of maternal mortality occurred in the postpartum period in 2019 alone [11].

Because they are more prone to iron deficiency and anemia during pregnancy due to dietary and physiological factors, breastfeeding moms are viewed as being more vulnerable than non-lactating mothers [12].

Iron deficiency anemia during the postpartum period can have very serious and long-term effects on mothers and their babies. Anemia is a contributing factor to maternal morbidity, mortality, and complications indirectly [7]. Anemia damages women’s health and well-being and raises the possibility of unfavorable results for mothers and newborns [13]. It represents 2% of Ethiopia’s overall maternal death rate [14]. A 10% g/l increase in maternal hemoglobin has been estimated to reduce maternal mortality by 29% and perinatal mortality by 28%. Anemia and mother death have a linear association [15]. Due to bleeding, postnatal moms lose a substantial amount of iron during birth; every milliliter of blood lost is equivalent to 0.5 milligrams of iron lost [4, 16]. It can cause morbidity throughout the reproductive cycle if it is not detected and treated early [16].

A major issue in public health is postpartum anemia. As a result of postpartum anemia, some moms experience despair, emotional instability, exhaustion, infection, and decreased quality of life. Additionally, their babies endure poor care and insufficient breast milk production [17].

Negative effects of postpartum anemia include reduced quality of life, dyspnea, palpitations, infections, exhaustion, altered cognitive function, unstable emotions, and postpartum depression. Therefore it has an impact on breastfeeding, caregiver capacity, and the attachment between a mother and her kid [5, 18]. Iron deficiency anemia in babies is increased by early weaning from breastfeeding, as breast milk itself has low iron content. Limited birth weight and preterm newborns have limited iron stores at birth, which is critical for development, immunity, and growth. As a result, babies exposed to infections experience poor growth and development in addition to morbidity and mortality [4, 19].

Preventing postpartum anemia is critical to the health of expectant mothers and their babies. Therefore, the WHO advises postpartum women to take oral iron supplements for three months following delivery, either with or without folic acid [17]. Additionally, weekly supplements of 60 mg iron and 2.8 mg folic acid for all women of reproductive age and 60 mg + 400 µg iron folic acid for expectant mothers were advised in populations where the prevalence of anemia was higher than 20% [20]. Even in Ethiopia, postpartum moms were not given IFA or evaluated for anemia in the research area. On the other hand, inadequate adherence poses a challenge to the emphasis placed on IFA supplementation during pregnancy. 5% of those who took iron supplements for 90 days or longer remained below the inadequate level, indicating poor iron use [7].

Dietary content and quantity have a significant impact on the amount of iron absorbed from prescribed iron folate or diet. It is challenging to improve maternal iron status and meet the second global nutrition target, which calls for a 50% reduction in anemia among reproductive-age individuals by 2025 unless iron supplementation is continued for periods ranging from 12 weeks to two years and “nutrition-specific interventions” are implemented for all women in the reproductive cycle [4, 20].

To lessen this issue, the WHO advises postpartum women to take iron supplements for six to twelve weeks following birth in areas where anemia during pregnancy is a public health risk [17]. However, in the study area, there is no postnatal anemia screening and iron supplementation. Poor adherence can lead to postpartum anemia by affecting the iron reserve from the recommended IFA during pregnancy and making it difficult to handle iron loss during childbirth [21]. The EDHS found that the prevalence of anemia among nursing moms increased by 10.6% between 2011 and 2016, despite a reduction goal of 18–12% [7, 8]. This discrepancy may be due to failure to emphasize during preconception and postnatal period. It has many contributions to offer evidence for policymakers and stakeholders, aiding in the development and implementation of evidence-based interventions to address the morbidity and mortality associated with anemia in postpartum women in Ethiopia.

## Methods

### Study design and search strategy

A systematic review and meta-analysis of published studies were used. Searching was made from beginning of January to end of January 2024.A comprehensive examination of all available research studies was conducted across major databases, including Google Scholar, PubMed, and the Cochrane Library. Additionally, efforts were made to retrieve new articles by reaching out to experts and researchers, and manual searches were performed to identify unpublished studies. The search utilized specific Keywords and MeSH terms such as “Anemia,” “low hemoglobin,” “Postpartum,” “post-natal women,” and “Ethiopia.” The Preferred Reporting Items for Systematic Reviews and Meta-analyses (PRISMA) guidelines will be adhered to meticulously during the course of this review.

### Study selection and eligibility criteria

This systematic review and meta-analysis encompassed cross-sectional studies conducted in Ethiopia. Eligible studies included those with primary research designs, while review articles, conference abstracts, and editorials were excluded. The criteria for inclusion also specified studies measuring the prevalence of anemia among postpartum women in Ethiopia between 2015 and 2023. The selection process adhered to the Preferred Reporting Items for Systematic Review and Meta-Analysis (PRISMA) guidelines. Two authors independently screened the studies for eligibility, and any disparities were resolved through discussion and consensus.

The protocol for this review has been prospectively registered with the International Prospective Register of Systematic Reviews (PROSPERO) under the registration number CRD42024505959.

### Data extraction process

A standardized data extraction format was created using Microsoft Excel for retrieving information from the chosen studies. The format included categories such as author details (name and year of publication), study year, study setting, study design, sample size, study population, sampling procedures, data collection procedures, and findings. Two authors independently carried out the data extraction, and the results were cross-checked for consistency. In instances of discrepancies, a thorough review of the articles was conducted, and any disagreements were resolved through verification and subsequent discussion.

### Outcome of interest

The systematic meta-analysis focused on the outcome variable of anemia in postpartum mothers. According to the World Health Organization (WHO) criteria, anemia is defined as a hemoglobin concentration below 12.0 g/dl, with severity categorized as mild, moderate, and severe. The cutoff points for these categories are 10.0–11.9 g/dl, 7.0–9.9 g/dl, and < 7.0 g/dl, respectively.

### Study quality and risk of bias

Two authors independently conducted a risk of bias assessment for the selected studies using the Hoy 2012 tool, which comprises ten criteria. These criteria encompass the representation of the population, sampling frame, methods of participant selection, non-response bias, data collection directly from subjects, acceptability of case definition, reliability and validity of study tools, mode of data collection, length of prevalence period, and appropriateness of the numerator and denominator. The tool divides into four items assessing selection and non-response bias, five items evaluating measurement bias, and one item addressing bias related to analysis and results reporting. Each criterion was evaluated as either low or high risk of bias, and the overall risk of bias for each study was determined based on the total score of high-risk items: low (≤ 2), moderate (3–4), and high (≥ 5).

We utilized the GRADE (Grading of Recommendations Assessment, Development, and Evaluation) tool to assess the level of certainty of evidence for the outcome. The GRADE quality evaluation tool initiates observational studies with a low quality of evidence, and this quality may further be downgraded to very low based on considerations such as risk of bias, inconsistency, indirectness, imprecision, and publication bias. However, there is an option for upgrading if no other limitations are identified within these factors. Assessments were conducted for the five primary domains (risk of bias, consistency, directness, precision, and publication bias), along with an evaluation of the overall quality of evidence. Following the GRADE recommendations, the study design served as the starting point, and for each domain not met, a one-step downgrade was applied [22].

The quality of studies was evaluated using the Joanna Briggs Institute (JBI) critical appraisal checklist. Following a protocol, the reviewers (GL, EKB) employed a blinded review approach to assess the quality of the original articles. Studies scoring 5 or more on the JBI criteria [23] were deemed to have good quality and were consequently included in the review. Any discrepancies in the quality assessment were resolved through consultation with the first author.

### Statistical analysis and synthesis

The collected data were inputted and analyzed using STATA version 17 statistical software. To calculate the variance of postpartum anemia prevalence for each article, the binomial distribution formula was applied by extracting the frequency of the outcome and the sample size. The random-effects model was used to calculate the pooled odds ratio (OR) with a 95% confidence interval, determining factors associated with anemia among postpartum women in Ethiopia. Heterogeneity among the studies was evaluated through the Cochran Q test (a *P*-value < 0.10 was considered significant) and I2 statistics (a significance level of at least 50%).

Estimation was conducted using a random-effects model with a 95% confidence interval (CI) due to notable variations among the study findings. The choice of a random-effects model, known for its conservative approach, was made to accommodate the inherent heterogeneity in meta-analysis. Subgroup analysis was carried out according to the study’s location. To identify publication bias, funnel plot analysis, Egger weighted regression, and Begg rank correlation tests were employed, with a significance level set at *P* < 0.05. The outcomes of the meta-analysis were visually presented through forest plots and tables.

## Result

### Characteristics of the studies

**Fig. 1:**
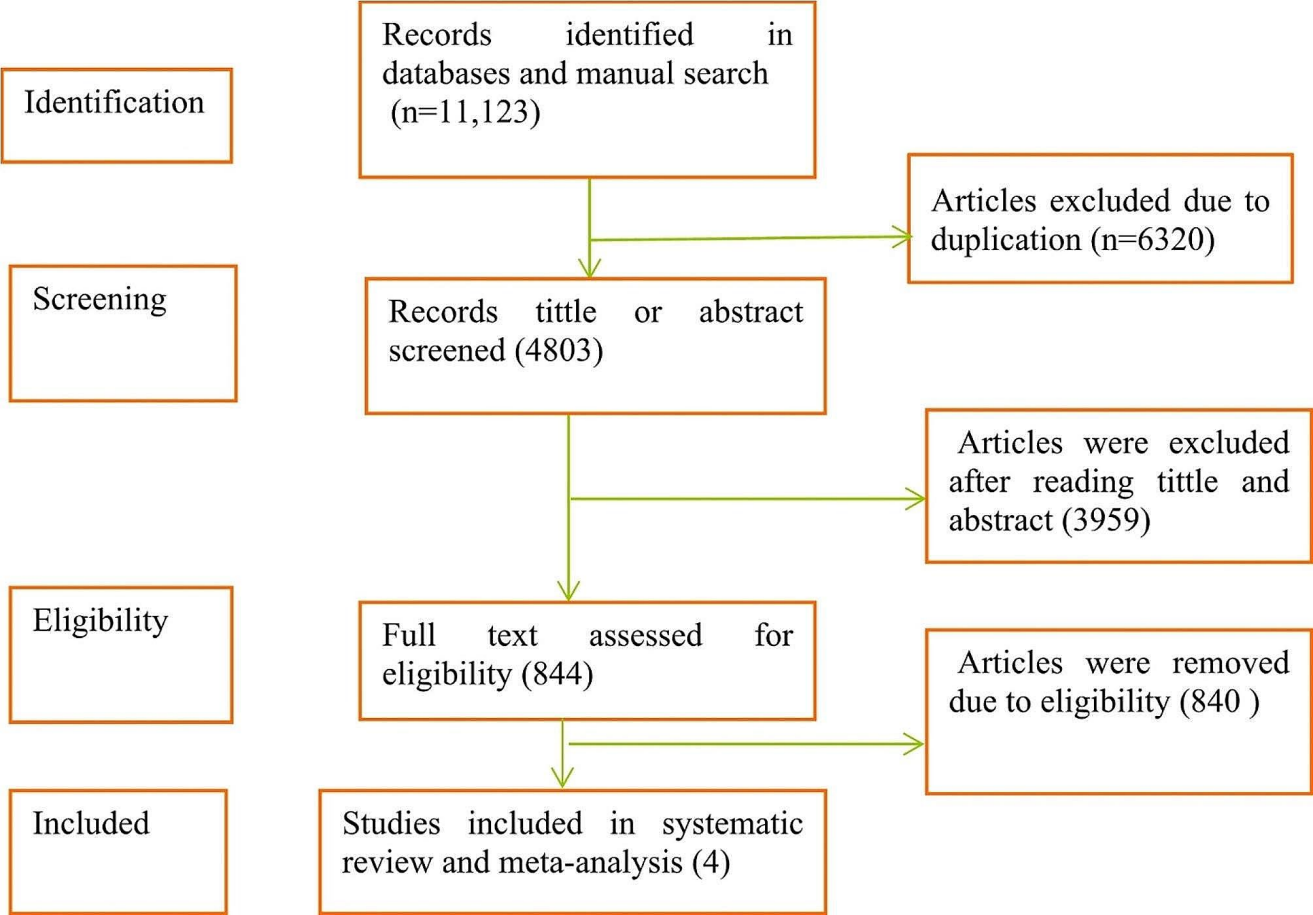
PRISMA flow chart diagram describing selection of studies for systematic review and meta-analysis on the prevalence of Anemia among postpartum women in Ethiopia, 2024

A total of 11,123 published studies were retrieved through searching from different databases. Out of 11,123 studies, 6320 studies were excluded due to duplication, and 3959 studies were excluded after reading the title and abstract using inclusion and exclusion criteria since they did not relate to the aim of this study. The remaining 844 full- articles were assessed for eligibility. Finally, 4 studies were included in the systematic review and meta-analysis (Fig. 1).All four studies included in a systematic review and meta-analysis were cross-sectional studies.

**Fig. 2:**
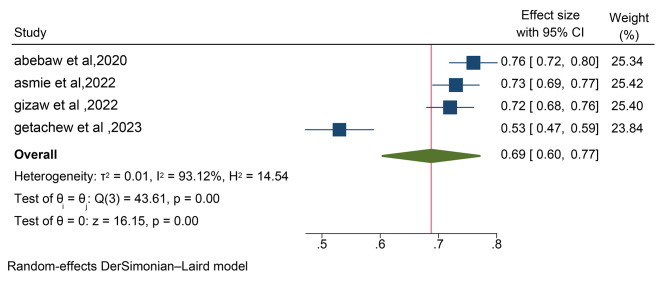
Forest plots of four studies on the prevalence of anemia among postpartum women in Ethiopia, 2024

**Fig. 3:**
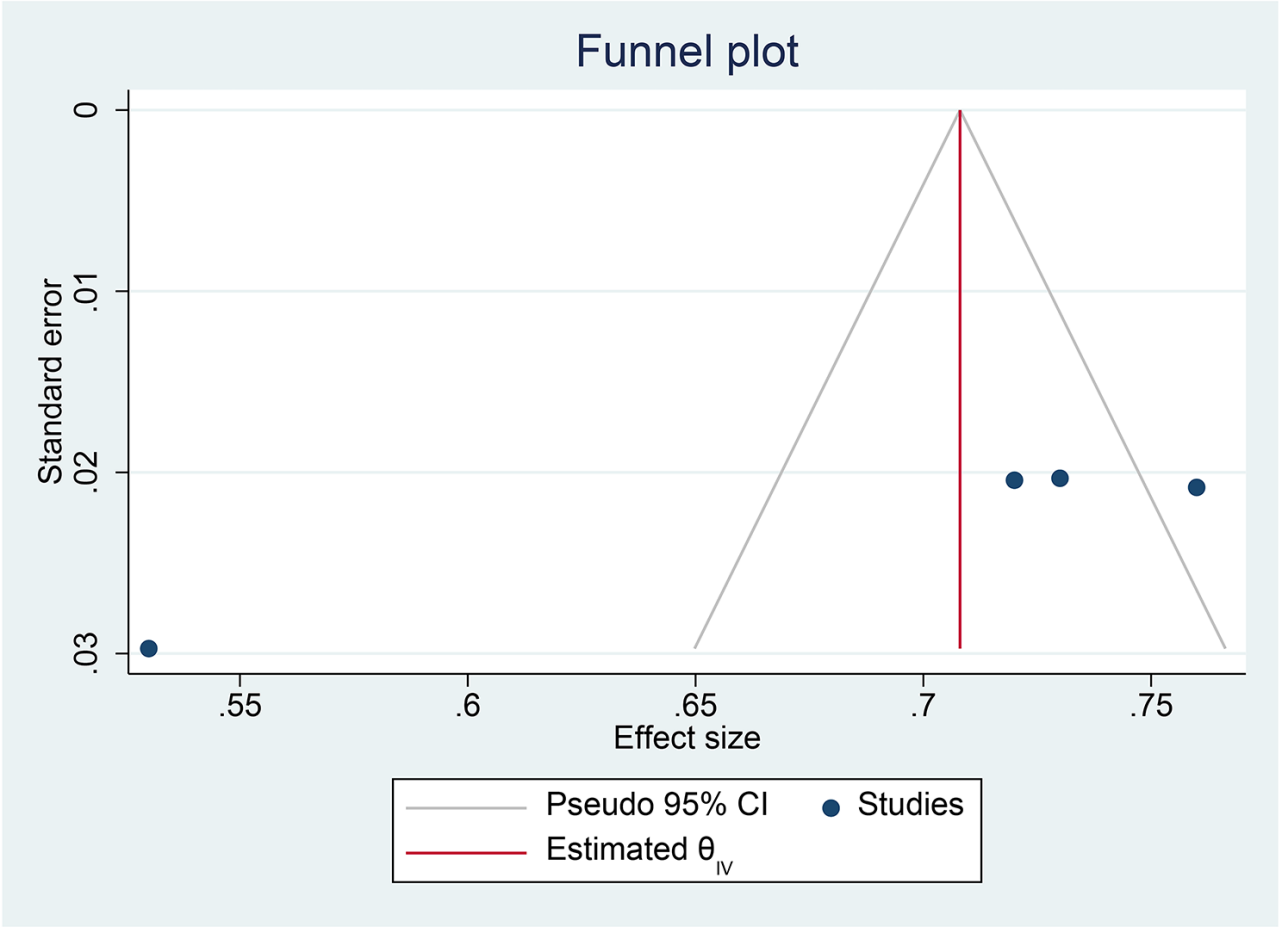
A Funnel plot of studies conducted on prevalence of anemia among post partum women in Ethiopia, 2024

**Fig. 4:**
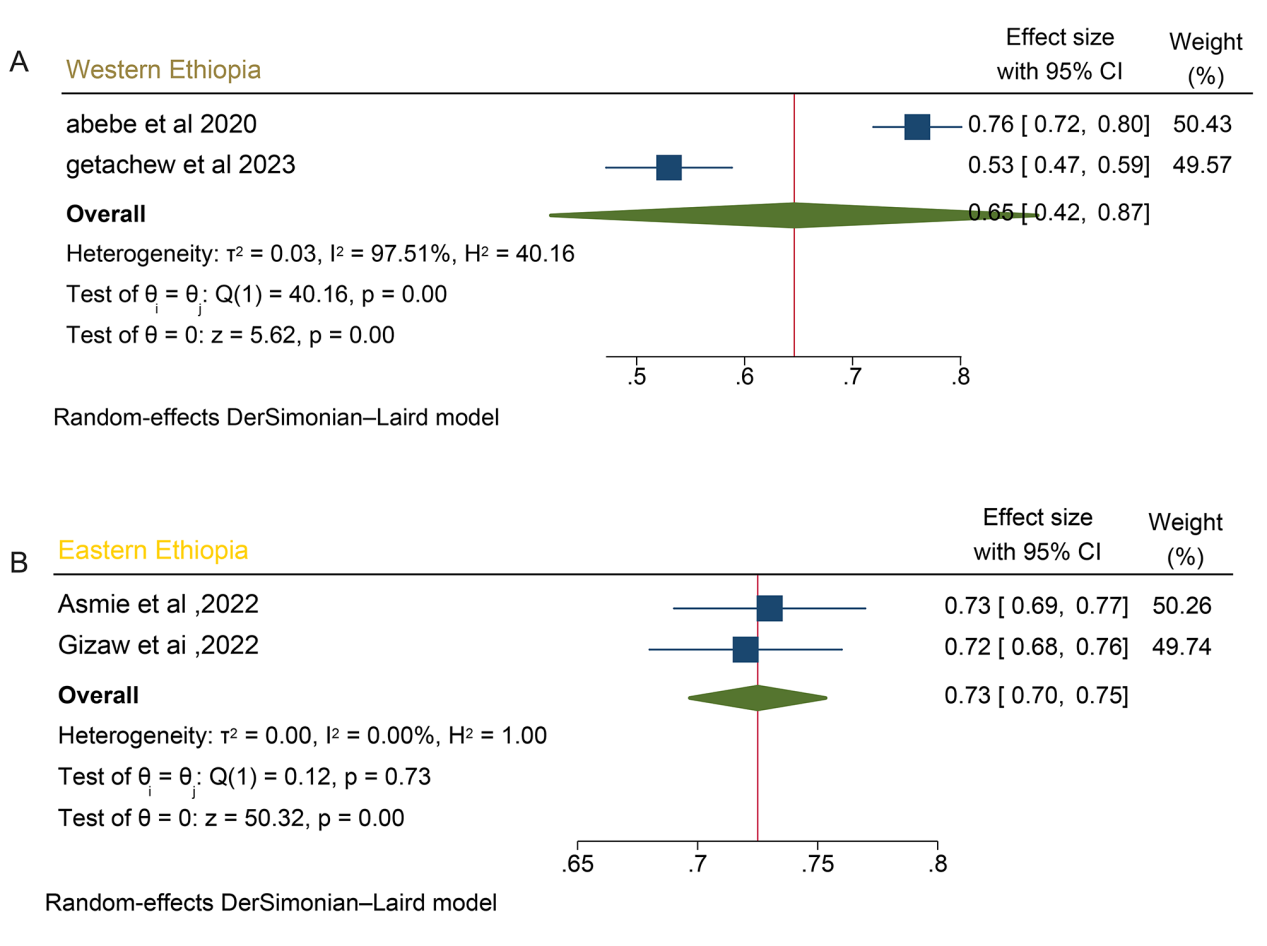
Sub group analysis based on place of study on prevalence of postpartum anemia in Ethiopia, 2024 **Fig A:** sub group analysis of western Ethiopia on postpartum anemia in Ethiopia **Fig B:** sub group analysis of eastern Ethiopia among postpartum anemia in Ethiopia

The assessment of bias risk for the four individual articles included in the systematic review and meta-analysis utilized the Hoy 2012 tool with ten specified criteria, as outlined in the methodology section. Among the four studies, two (50%) were identified as having a low risk of bias, while the remaining two studies (50%) were categorized as having a moderate risk of bias. These four studies, which examined the prevalence of anemia in postpartum women, demonstrated significant heterogeneity, as indicated by the Cochrane Q test (*p* = 0.00) and I2 test (93.1%), warranting the use of a random-effects model (Fig. 2). However, the Egger weighted regression statistics for studies on anemia prevalence (*P* = 0.00) and Begg rank correlation statistics (*p* = 0.0) revealed evidence suggesting the presence of publication bias. The funnel plot has also an asymmetry by visual inspection also shows there is a sign of publication bias (Fig. 3). To decrease the heterogeneity, subgroup analysis was performed based on the region (Tables 1 and Fig. 4). More over to treat publication bias we run non parametric trim-and-fill analysis, however no imputed studies observed.

**Table 1:** Characteristics of the four studies included in systematic review and meta-analysis

Author	Survey year	Place of the study	Study setting	Sample size	Sampling procedure	Prevalence of anemia	JBI score	Ref
Abebaw	2020	Debremarkos	Facility based cross-sectional	424	Systematic random sampling	24.3%	8	[24]
Asima	2022	Dire-Dawa	Facility based cross-sectional	476	Systematic random sampling	26.9%	7	[25]
Gizawu	2022	Harer	Facility based cross-sectional	484	Systematic random sampling	28.1%	9	[26]
Getachew	2023	Gonar town	Facility based cross-sectional	282	Systematic random sampling	47.1%	8	[1]

**Table 2:** Subgroup analysis of the prevalence of postpartum anemia by place of study using I2 test for heterogeneity, 2024

Subgroup	Anemia prevalence	I 2 (%)	*P*-value
Eastern Ethiopia (Diredawa and Harer)	72.5%; CI (0.697, 0.753)	0	0.0000
Western Ethiopia (Debremarkos and Gondar)	64.6% ;CI (0.421,0.871)	97.5	0.0000

### Prevalence of anemia among postpartum women in Ethiopia

A total of four studies were included in this systematic review and Meta-analysis to reveal the prevalence of anemia in postpartum women in Ethiopia from 2015 to 2023. A study done in 2020 reported there was a 24.3% prevalence of anemia among postpartum women in the Amhara region in Debre Markos. Whereas in a study conducted in 2022, the prevalence of anemia in postpartum women was 26.9% and 28.1% in Diredawa and Harer respectively. Another study conducted in the Amhara region, Gondar documented a relatively high prevalence (47.1%) of anemia in postpartum women in 2023 (Table 2).

The pooled prevalence of anemia among postpartum women in Ethiopia was 69% (95%CI: 60- 77%) (Fig. 2).

Furthermore, subgroup analysis based on the place of study showed that the prevalence of anemia in postpartum women was significantly higher (72.5% ) in Eastern Ethiopia (Diredewa and Harer) compared to the lowest prevalence ( 64.6%) in Western Ethiopia (Gondar and debremarkos (Table 1).

### Meta-regression and sensitivity analysis

#### Meta-regression

Meta-regression was performed with the place of study considered as covariates, employing a random-effects model. The outcome indicated the absence of heterogeneity based on the place of study (*p* = 0.445) (Table 3).

**Table 3:** Meta-regressions of post-partum anemia by place of study of included studies in Ethiopia, 2024

Covariate	β (95% CI)	*p*-value
Place of study	0.0775 (− 0.1212365–0.2762696)	0.445

### Sensitivity analysis

**Fig. 5:**
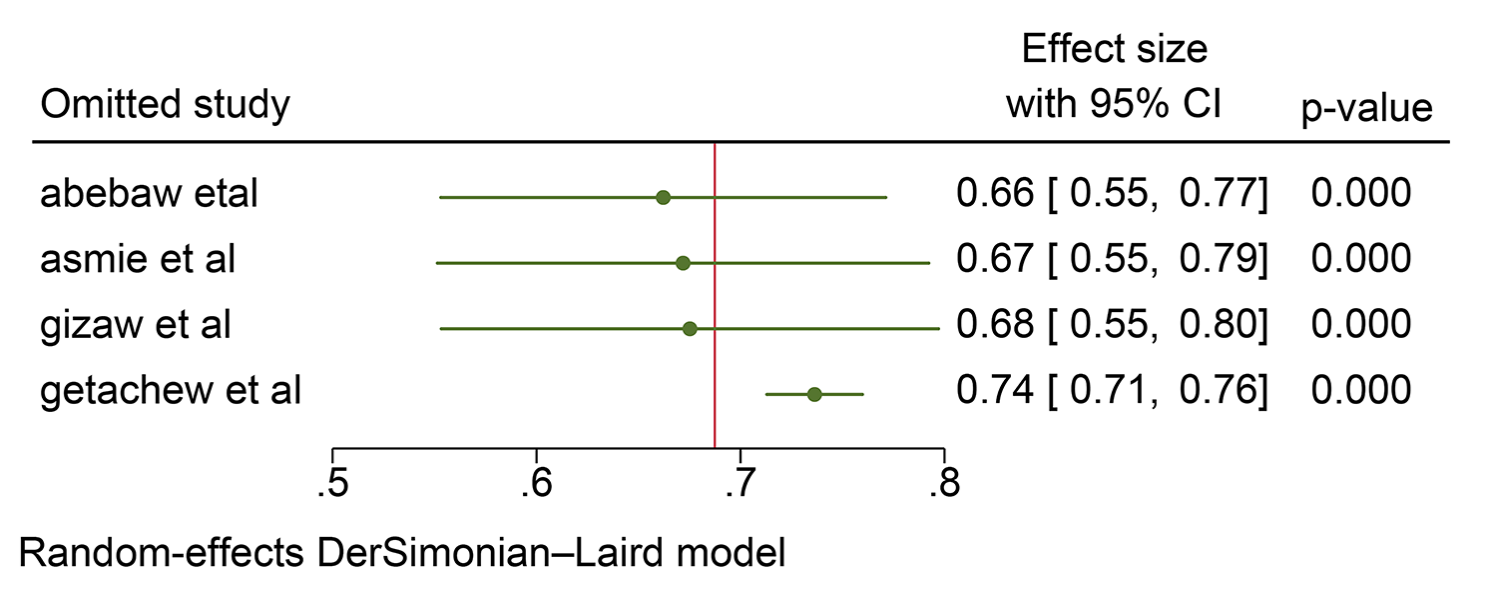
Leave-one-out sensitivity analysis of prevalence of postpartum women in Ethiopia, 2024

A sensitivity analysis was carried out using the leave-one-out method to evaluate the impact of each individual study on the overall pooled prevalence of postpartum anemia. The findings indicated that the estimated prevalence obtained when each study was excluded from the analysis fell within the confidence interval of the pooled prevalence. Consequently, none of the included studies had a significant effect on the overall pooled estimate, as demonstrated below (Table 4 and Fig. 5).

**Table 4:** sensitivity analysis on prevalence of post-partum anemia in Ethiopia, 2024

Study	effect size	[95% conf. Interval]		% weight
Abebaw et al. 2020	0.760	0.719	0.801	25.34
Asmie et al. 2022	0.730	0.690	**0.770**	**25.42**
Gizaw et al. 2022	0.720	0.680	0.760	25.40
Getachew et al. 2023	0.530	0.472	0.588	23.84
theta	0.687	0.604	0.771	

95% prediction interval for theta: [0.290, 1.085]

### Factors associated with Anemia among postpartum women in Ethiopia

Before doing the pooled associated factors, there were twelve associated factors for post partum anemia : Lack of formal education [25, 26], ANC visit < 4 [24–26] ,history of PPH [1, 24] ,history of APH [24], C/S [1, 26] ,vaccum/forceps [24, 25], pre delivery Hgb < 11gm/dl [25, 26], poor adherence to IFA supplementation [1, 24–26] ,low diet [1, 25] ,maternal blod loss [26] ,MUAC < 23 cm [24], GIT [25] and unemployment [25] were the independent variables associated with post partum anemia .

Lack of formal education was one of the socio economic factor that has significantly associated with post partum anemia.Women who lacked formal education were 3.5 times more likely to be anemic than their educated counter parts (OR = 3.5; 95% CI: 2.639, 4.408).

ANC visit less than 4, history of PPH, history of Forceps/vaccum delivery, pre delivery Hgb less than 11gm/dl and history of cesarean delivery are obstetric factors that are significantly associated with post partum anemia .Women who had lower antenatal care visits (below 4) (OR = 2.72; 95% CI:2.14, 3.3) had 2.72 times more likely odds of developing anemia compared to their respective counterparts. Mothers who had history of PPH had 2.49 times higher odds of anemia than those that had not history of PPH (OR = 2.49, CI:1.075,3.978). Women who had forceps/vaccum delivery were 3.96 times more likely to be anemic than their counter parts (OR = 3.96; 95% CI: 2.986, 4.947).Mothers who had also pre delivery Hgb less than 11gm/dl were 4.2 times more likely to be anemic than those who had normal pre delivery Hgb (OR = 4.2CI:1.768,6.668). Women who delivered via C/S are 4.4 times at higher risk of developing anemia compared with their SVD counter parts.

The meta-analysis also showed that poor adherence for iron and folic acid supplementation during pregnancy and having low diet are the nutritional factor that were significantly associated with post-partum anemia. Mothers who had poor adherence for iron and folic acid supplementation are also 2.8 times more likely to be anemic than their counter parts (OR = 2.8 CI:2.311,3.297).Women with a low level of dietary diversity were 2.45 times more likely to suffer from anemia than those with a minimum level of dietary diversity (OR = 2.45 CI :1.214–3.703) (Table 5**).**

**Table 5:** The pooled odds ratios of factors associated with anemia among postpartum women in Ethiopia, 2024

Factor Variables	Odds Ratio (95% CI) (Random effect model)	I 2(%)	*P*-value
ANc visit < 4 times	2.72(2.14–3.3)	26.85	**0.0000**
PPH	2.49(1.075–3.978)	20.43	**0.0006**
Forceps/vaccum	3.96(2.986–4.947)	0.000	**0.0000**
IFA	2.8 (2.311–3.297)	0.00	**0.0000**
Lack of formal education	3.5 (2.639–4.408)	52.37	**0.0000**
Low Pre delivery Hgb	4.2(1.768–6.668)	96.69	**0.0007**
Low diet	2.45(1.214–3.703)	89.18	**0.0001**
Ceaserean delivery	4.04(3.426–4.671)	0.00	**0.0000**

## Discussion

This systematic review and meta-analysis compiled evidence on the prevalence and factors associated with anemia among postpartum women in Ethiopia in the year 2024.

The pooled prevalence of anemia among post-partum women in Ethiopia was found to be 69%. This discovery exceeds the results of a study carried out by the Ethiopian Demographic and Health Survey (EDHS), which reported a 28.6% prevalence of anemia among postpartum women in the country in 2016 [27]. This could be linked to the comparatively elevated prevalence of infectious diseases and the presence of vulnerable health institutions [27] and this finding is also higher than studies conducted in Germany(22%) [28], Kenya(16.4%) [29]. The variation observed may be attributed to differences in the timing of the studies and certain Sociodemographic factors such as age and educational attainment. Additionally, such discrepancies could arise from variations in sample sizes and the utilization of different time frames for postpartum anemia assessment [27].

Subgroup analysis based on place of study indicated that significantly higher (72.5%) prevalence of anemia in Eastern Ethiopia (Harer and Dire dawa) compared with 64.6% in studies conducted in Western Ethiopia (Gondar and Debremarkos). The disparities in anemia prevalence across the regions may be linked to regional variations in food consumption preferences [30, 31], the rate of infectious disease occurrence [31] and variation in healthcare services accessibility [27]. The differences across study locations emphasize the significance of disaggregated data for informed policymaking and program development. Context-specific interventions are necessary to address anemia in Ethiopia.

Post-partum women’s educational status was significantly associated with anemia. Women who had formal education had a significantly lower likelihood of developing anemia than educated counter parts. This finding is consistent with other studies from developing countries [32]. This is because women with higher education are more inclined to utilize healthcare services and have a more diverse diet compared to mothers with lower education levels [27].

Women who had received the recommend at least four antenatal care visits were found to be less likely to develop anemia during the postpartum period. This could be attributed to the provision of education on nutrition and health, particularly emphasizing the importance of including diverse sources of iron-rich foods to alleviate anemia [27].

Women who did not receive iron-folate supplementation during their latest pregnancy were susceptible to the onset of postpartum anemia. This finding is in consistent with studies from countries such as Karachi, Pakistan [33], and India [34]. One plausible explanation is that iron serves as a vital replenishment for blood loss and tissue growth during pregnancy and childbirth, given the heightened physiological demands and depletion of iron during these phases [35]. Research suggests that the intake of a minimum of 90 iron-containing tablet supplements during pregnancy has the potential to decrease maternal anemia by as much as 70% [36]. Another reason could be that women who initially have iron deficiency without anemia early in pregnancy may develop anemia later due to reduced or ineffective erythrocyte production, leading to immediate postpartum anemia [37].

The odds of developing immediate PPA increased among women who gave birth via cesarean section compared with those who gave birth via SVD. This result aligns with earlier studies conducted in various countries. Such as Jeddah, Saudi Arabia [38], and Pakistan [33]and developed countries like Madrid, Spain [10], and Bursa, Turkey [39]. One potential explanation is that women who have experienced a cesarean section may be more prone to postpartum hemorrhage (PPH), leading to a reduction in red blood cell (RBC) production and an increase in nutrient losses due to bleeding [40]. Another proposition might involve uterine atony resulting from prolonged labor, uterine tears, lacerations due to obstructed labor, and retroplacental clot formation following placental abruption. These factors lead to severe bleeding by impeding uterine contraction, and they collectively serve as indications for a cesarean delivery [41].

Pre delivery hemoglobin levels less than eleven were another independent factor strongly associated with PPA during the immediate postpartum period. This finding is in line with different studies conducted in developed countries such as [34]Spain [10], and Tamil Nadu, India [34]. The potential reasons could include a pre-delivery low hemoglobin (Hgb) level, reduced myometrial contractility, and compromised coagulation due to the impaired transport of Hgb and oxygen to the uterus. This situation leads to tissue enzyme and cellular dysfunction, ultimately resulting in uterine atony, which stands as the most prevalent cause of postpartum hemorrhage (PPH) (53).

The odds of anemia among postpartum mothers who experienced massive postpartum blood loss were 2.49 times higher than the odds of anemia among postnatal mothers who did not develop postpartum haemorrhage. These similar findings were conducted in Saudi Arabia, and Tamil Nadu, India [32, 36].

Mothers who gave birth by instrumental (vacuum or forceps) assisted mode of delivery were almost 3.96 times more likely to be anemic in the postpartum period when compared to those who gave birth through spontaneous vaginal delivery. This finding were consistent with the studies done in Spain (two studies) and Saudi Arabia [10, 37]. This could be attributed to the fact that instrumental assisted vaginal delivery heightens the likelihood of episiotomy, spontaneous perineal, and/or cervical tears, which may also extend to the uterus. Clinicians often misdiagnose these tears and perform repairs after significant bleeding occurs in mothers.

Maternal nutritional status was found to be significantly associated with anemia. Women with a low level of dietary diversity were 2.4 times more likely to suffer from anemia than those with a minimum level of dietary diversity. This finding was consistent with a study conducted among lactating women in Jimma District [42]. This may stem from insufficient dietary intake, resulting in deficiencies of iron, vitamin B12, folate, and vitamin A. Another factor could be a deficiency in protein and foods containing iron [43].

### Implications of the study

This research aimed to ascertain the collective prevalence and factors linked to anemia in postpartum mothers in Ethiopia. The objective was to offer evidence for policymakers and stakeholders, aiding in the development and implementation of evidence-based interventions to address the morbidity and mortality associated with anemia in postpartum women in Ethiopia.

### Strength and limitations of the study

Thorough searches were conducted using various strategies, both manual and electronic, to include a range of published and unpublished articles. To mitigate bias, two authors independently extracted data using a predefined tool, and one author performed a quality assessment. Addressing high heterogeneity, subgroup analysis and the random-effects model were employed to calculate the pooled prevalence and odds ratio.

Potential biases, such as inaccurate selection of study participants, small sample sizes in some studies, limitations in data collection and analysis, and selective reporting of results in the included studies, could impact the findings of the meta-analysis. The cross-sectional design of the original studies in this review raises the possibility of confounding variables influencing the estimates. Unexamined confounders may contribute to the heterogeneity observed in the prevalence of anemia among the studies reviewed.

## Conclusion

The study indicated that anemia in post-partum women is a major public health problem in Ethiopia. ANC visit less than 4, history of PPH, vaccum/forceps delivery, poor adherence to Iron and folic acid supllemtation during pregnancy, low diet, predelivery hemoglobin less than 11, history of cesearn delivery and lack of eduction were factors associated with higher odds of developing anemia in post-partum women in Ethiopia. Therefore, it is imperative to integrate health education and promote the usage of iron and folate supplements during pregnancy, along with encouraging dietary diversity practices. Additionally, it is crucial to align these interventions with women’s sustained income-generating activities. Preventing anemia in women who undergo cesarean deliveries entails ensuring efficient CS delivery, promoting a positive long-term health outlook after CS, and implementing postoperative monitoring. Hence, focused attention is essential to ensure effective antepartum, intrapartum, and postpartum maternal care.

The government of Ethiopia also needs to monitor and evaluate the implementation and effectiveness of nutrition programs in Ethiopia in order to strengthen, design, and effectively implement comprehensive multi-sectorial community and facility-based interventions like micronutrient supplementation, and nutrition education in order to prevent and reduce anemia morbidity among post-partum women in Ethiopia.

## Data Availability

All the data included in systematic review and Meta-analysis are available in the main manuscript.
