# Correction: Prevalence and associated factors of anemia among postpartum mothers in public health facilities in Ethiopia, 2024: a systematic review and meta-analysis

**DOI:** 10.1186/s12884-024-06781-9

**Published:** 2024-08-30

**Authors:** Gebeyehu Lakew, Amlaku Nigusie Yirsaw, Alemshet Yirga Berhie, Asnake Gashaw Belayneh, Solomon Ketema Bogale, Eyob Getachew, Getnet Alemu Andarge, Kedir Seid, Eyob Ketema Bogale

**Affiliations:** 1 Health Promotion and Communication Department, School of public health, College of medicine and health sciences, Gondar University, Gondar, Ethiopia; 2 Nursing department, college of medicine and health science, Bahir Dar University, Bahir Dar, Ethiopia; 3 Department of emergency and critical care nursing, College of Medicine and Health Science, Bahir Dar University, Bahir Dar, Ethiopia; 4 Department of Nutrition, Antsokiya Gemza wereda Health Office, North Shoa, North East, Ethiopia; 5 Bati Primary Hospital, Oromia Special Zone, North Shoa, North Central, Ethiopia; 6 Health Promotion and Behavioral science department, school of public health, College of medicine and health science, Bahir Dar University, Bahir Dar, Ethiopia

**Correction**: ***BMC Pregnancy Childbirth* 24**,** 327 (2024)**

10.1186/s12884-024-06525-9

Following publication of the original article [1], the authors identified an error in the author name of Amlaku Nigusie Yirsaw (use the correct updated name).

The incorrect author name is: Amlaku Nigussie Yirsaw.

The correct author name is: Amlaku Nigusie Yirsaw.

The author group has been updated above and the original article [1] has been corrected.
